# Understanding the Binding Induced Folding of Intrinsically Disordered Proteins by Protein Engineering: Caveats and Pitfalls

**DOI:** 10.3390/ijms21103484

**Published:** 2020-05-15

**Authors:** Francesca Malagrinò, Lorenzo Visconti, Livia Pagano, Angelo Toto, Francesca Troilo, Stefano Gianni

**Affiliations:** Istituto Pasteur—Fondazione Cenci Bolognetti, Dipartimento di Scienze Biochimiche ‘A. Rossi Fanelli’ and Istituto di Biologia e Patologia Molecolari del CNR, Sapienza Università di Roma, 00185 Rome, Italy; francesca.malagrino@uniroma1.it (F.M.); lorenzo.visconti@uniroma1.it (L.V.); livia.pagano@uniroma1.it (L.P.); angelo.toto@uniroma1.it (A.T.); francesca.troilo@uniroma1.it (F.T.)

**Keywords:** IDP, Φ value analysis, protein engineering, disorder-to order transition, folding kinetics

## Abstract

Many proteins lack a well-defined three-dimensional structure in isolation. These proteins, typically denoted as intrinsically disordered proteins (IDPs), may display a characteristic disorder-to-order transition when binding their physiological partner(s). From an experimental perspective, it is of great importance to establish the general grounds to understand how such folding processes may be explored. Here we discuss the caveats and the pitfalls arising when applying to IDPs one of the key techniques to characterize the folding of globular proteins, the Φ value analysis. This method is based on measurements of the free energy changes of transition and native states upon conservative, non-disrupting, mutations. On the basis of available data, we reinforce the validity of Φ value analysis in the study of IDPs and suggest future experiments to further validate this powerful experimental method.

## 1. Introduction

Whilst the classical view of molecular biology is intimately linked to the structure-function dogma, it is now widely accepted that a large fraction of the proteome lacks a well-defined three-dimensional structure [1,2,3,4]. These systems, typically denoted as intrinsically disordered proteins (IDPs), exert a variety of important functions within the cellular environment, despite being essentially unstructured at physiological conditions [5,6,7,8,9,10]. Because of their relevance and abundance, intense research is currently devoted to understanding the molecular functions of IDPs. Among the open questions, it is of particular interest to clarify the role of disorder, if any, in mediating the physiological behaviours of IDPs, in comparison to those of more structured proteins.

A recurrent feature of many IDPs lies in their ability to gain a more ordered structure when binding to their physiological partner(s) [11,12,13,14,15]. This process, generally referred to as ‘induced folding’, may however vary extensively between different IDPs, such that whilst some proteins display a pronounced disorder-to-order transition [16], in other cases the resulting complex may retain a considerable amount of disorder [17,18]. A general concept describing these different scenarios, the so-called ‘fuzziness’, has been introduced by Fuxreiter and co-workers, who provide a comprehensive quantitative description of these different phenomena; for further reading, refer to [19,20,21]. Whilst it is clear that the level of disorder in different complexes involving IDPs may vary considerably, it may be postulated that in all cases binding results in changes in the structural and dynamic properties [22]. Therefore, the study of the mechanisms whereby IDPs undergo disorder-to-order transitions represents a critical piece in the puzzle of their understanding and characterization.

In the case of globular proteins, much of our current knowledge on folding has been acquired by taking advantage of the synergy between site-directed mutagenesis and kinetic studies [23]. This method, known as ‘Φ value analysis’ and extensively discussed below, allows mapping the structure of meta-stable state(s) along reaction pathways and has a terrific impact on both experimental and computational work on protein folding over the past decades. Because of its power, the Φ value analysis is a very appealing technique also in the study of disorder-to-order transitions of IDPs [11,15,24,25,26,27,28,29,30,31,32,33,34,35]. Yet, there might be caveats and pitfalls that should be carefully considered. In this review, we recapitulate the key milestones and advices on the Φ value analysis, put them in the context of the possible complications arising in the case of IDPs and discussed them in the light of the work already present in the literature.

## 2. Depicting Reaction Mechanisms at Nearly Atomic Resolution—Design and Principles of Φ Value Analysis

One of the most ambitious goals of the biophysicist is to provide a structural depiction of reaction mechanisms at nearly atomic resolution. However, in the case of protein folding, this is particularly challenging. In fact, because the reaction typically occurs in a highly cooperative manner, it is very difficult to capture any snapshots between the unfolded and the folded states, with the apparent transition often resembling an all-or-none two-state behavior [36]. Importantly, whilst it would be limited, if not wrong, to exclude the presence of intermediates in protein folding, we note that these states are typically very elusive and often accumulate only transiently [37]. Consequently, for the sake of simplicity, we will first focus on two-state folding, even though all the considerations that are made below regarding the transition state can be potentially extended to any metastable state along the folding reaction, such as intermediate states [38].

**Figure 1:**
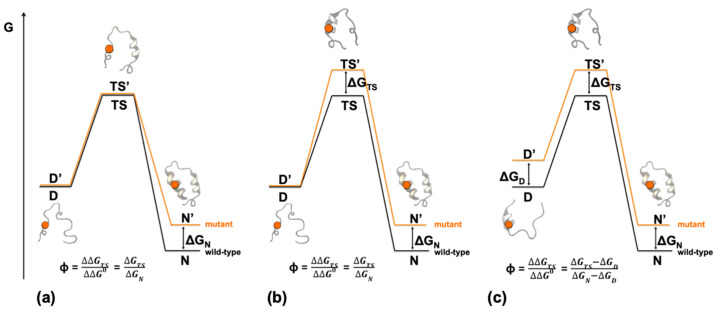
Different Φ value profiles and related free energy diagrams for a two-state folding reaction of a wild-type (black line) and mutated (orange line) protein. The mutation is highlighted on the structure with an orange circle. ΔG_N_, ΔG_TS_ and ΔG_D_ refer to the changes in stability upon mutation of the native, transition and denatured states respectively. (**a**) Φ value = 0. Mutation does not affect the structure and the stability of the transition state. The mutated region appears unstructured in the transition state likewise in the denatured state (ΔG_TS_ = 0) (**b**) Φ value = 1. The mutation insists in a native-like structured region of the transition state, with ΔG_TS_ tending to ΔG_N_. In both cases, the free energy of the denatured state does not change upon mutation (ΔG_D_ = 0). (**c**) The mutation affects the stability of the denatured state. This effect may reflect in an unusual Φ value, i.e., >1 or <0.

In a two-state reaction, the only experimentally accessible state, between the fully denatured and fully native states, is the transition state. Since the transition state never accumulates, information about its structure must be inferred indirectly [39]. The only experimental method to address the structural features of the transition state in protein folding is represented by the so-called Φ value analysis [23]. This method is based on the synergic usage of site-directed mutagenesis and kinetic (un)folding experiments. In particular, by systematically mutating side-chains in a protein while probing the effect of the variation on the native and transition states, it is possible to detect interaction patterns of the latter state. Quantitatively, the Φ value represents a structural index, which is calculated by normalizing the change in free energy upon mutation of the transition state to that of the native state. Thus, a Φ value may be calculated as follows:

(1) $$\Delta \Delta G_{D-N}=\Delta G_{D\prime -N\prime }-\Delta G_{D-N}$$

(2) $$\Delta \Delta G_{D-TS}=\Delta G_{D\prime -TS\prime }-\Delta G_{D-TS}$$

(3) $$\Phi =\frac{\Delta \Delta G_{D-TS}}{\Delta \Delta G_{D-N}}$$

where then symbol ‘ denotes the mutant protein. By following Equation (3), it may be concluded that if the site of mutation is native-like in the folding transition state, ΔΔG_TS-D_ tends to ΔΔG_D-N_ and Φ = 1. Conversely, if the site of mutation is denatured-like in the transition state, ΔΔG_TS-D_ tends to 0 and Φ = 0. Fractional Φ values are very frequently observed and represent a fractional formation of native-like structure in the transition state [23]. A pictorial representation of the underlying principle of Φ value analysis is reported in Figure 1.

Aside from the relatively simple theoretical basis of Φ value analysis, there are several practical caveats that demand additional consideration. Of particular importance, the mutations must be chosen properly. In fact, when a side-chain is mutated, the free energy is perturbed by mainly three components [23]: ΔG_solv_, arising from the change in solvation; ΔG_noncov_, arising from the perturbation of non-covalent interactions; and a ΔG_reorg_, which may be due to a structural reorganization upon mutagenesis. Since the Φ value analysis is aimed at detecting non-covalent formation of a native like structure in metastable states, the two terms ΔG_reorg_ and ΔG_solv_ should be held as low as possible. Consequently, Φ value analysis should be performed by using mostly aliphatic to aliphatic deletion mutations. Mutations of a large hydrophobic side chains may lead to very large ΔG_reorg_ and ΔG_solv_ and are therefore not suited for Φ value analysis. In conclusion, the recommended strategy is to mutate hydrophobic side chains, possibly by introducing minor truncations, without altering the stereochemistry (i.e., Ile→Val→Ala→Gly; Leu→Ala→Gly; Thr→Ser; Phe→Ala→Gly) [23]. On a general ground, it is generally helpful to produce and analyze a large number of site-directed mutants to substantiate any conclusion drawn from Φ value analysis. Because IDPs are generally depleted of hydrophobic amino acids in favor of polar and charged residues [40], it is more difficult to strictly follow these rules in this class of proteins. Hence, in those cases, to perform as many mutants as possible might be even more critical to provide a complete Φ value analysis.

## 3. The Φ Value Analysis in IDPs

The invention of Φ value analysis has revolutionized our understanding of protein folding and, after its introduction, has been employed in numerous folding studies. Furthermore, experimentally determined Φ values have been employed in benchmarking theoretical hypotheses, as well as in interpreting molecular dynamics simulations [41]. Given this critical importance, it is not surprising to observe that several laboratories are currently subjecting the binding-induced folding reaction of IDPs to the Φ value analysis. But how to calculate Φ values for IDPs?

In globular folding studies, the Φ value is defined as the ratio between the changes in activation free energy to that of the ground state upon mutagenesis (Equation (3)). Consequently, taking advantage of kinetic folding and unfolding experiments, the Φ value may be calculated as follows:

(4) $$\Delta \Delta G_{D-TS}=RT\ln\frac{k_{F}}{k\prime_{F}}$$

(5) $$\Delta \Delta G_{D-N}=RT\ln\frac{k_{F}}{k\prime_{U}}\frac{k\prime_{U}}{K\prime_{F}}$$

where *k_F_* and *k_U_* denote the folding and unfolding rate constant, respectively, and the symbol ‘ refers to a mutant protein, and then applying Equation (3).

In the case of IDPs, the folding reaction generally occurs concurrently with a binding event [12,14,15,22,27,32,33]. Therefore, it is very difficult to measure independently the microscopic folding and unfolding rate constants. To a first approximation, a Φ value analysis can only be carried out by measuring the overall association and dissociation rate constants under the so-called pseudo-first-order conditions. In practice, these types of experiments can be carried out by challenging one of the reactants, at lower concentration, with a different concentration of the interacting partner, at much higher concentration. Under this condition, the apparent rate constant of the reaction will be equivalent to:

$$k_{obs}=k_{on}\left[A\right]+k_{off}$$

where A is the reactant at higher concentration, *k_on_* and *k_off_* represent the microscopic association and dissociation rate constants, respectively, and *k_obs_* is the observed rate constant. In analogy with Equations (3)–(5), the Φ value analysis on an IDP can be carried out as follows

(6) $$\Delta \Delta G_{Free-TS}=RT\ln\frac{k_{on}}{k\prime_{on}}$$

(7) $$\Delta \Delta G_{Free-Bound}=RT\ln\frac{k_{on}}{k\prime_{off}}\frac{k\prime_{off}}{K\prime_{on}}$$

(8) $$\Phi =\frac{\Delta \Delta G_{Free-TS}}{\Delta \Delta G_{Free-Bound}}$$

It should be noticed, however, that the apparent *k_on_* and *k_off_* from pseudo-first-order binding experiments may in fact report of both the folding and binding events and their interpretations might thus not be straightforward. In an effort to help the reader in this perspective, a brief summary of the different scenarios is reported in the next section. Rarely, different kinetic phases may be detected, a condition that allows calculating the Φ values on sequential steps along the pathway. These cases are discussed for example in [24,25,42].

## 4. On the Cooperative Nature of Induced Folding and the Φ Value Analysis in IDPs

**Scheme 1:**
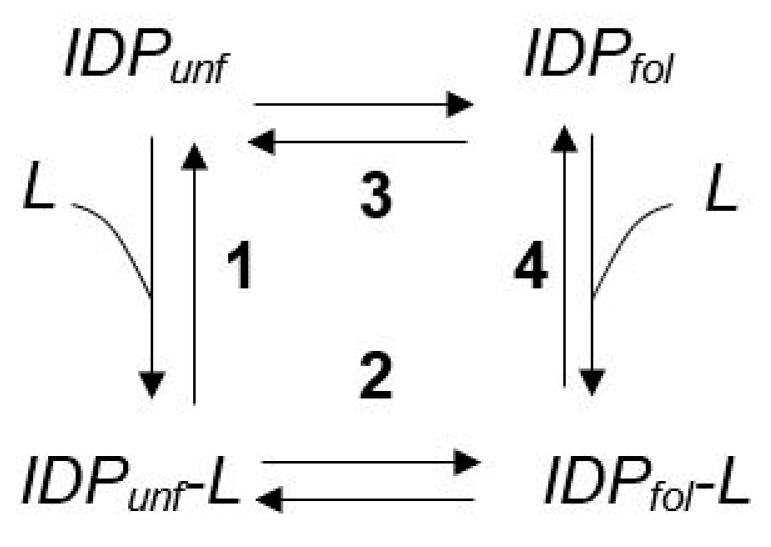
Schematic representation of the overall reaction mechanism involving the binding between an IDP and its ligand.

Because binding-induced folding is a complex reaction involving different steps, the interpretation of measured Φ values may be more complex than in globular folding. In fact, despite the observed reaction involves a folding and a binding step, induced folding can generally appear as simple as two-state; it should be noted that a comprehensive scenario describing an IDP undergoing a ligand-induced conformational change should be rigorously described by a squared Scheme 1.

**Figure 2:**
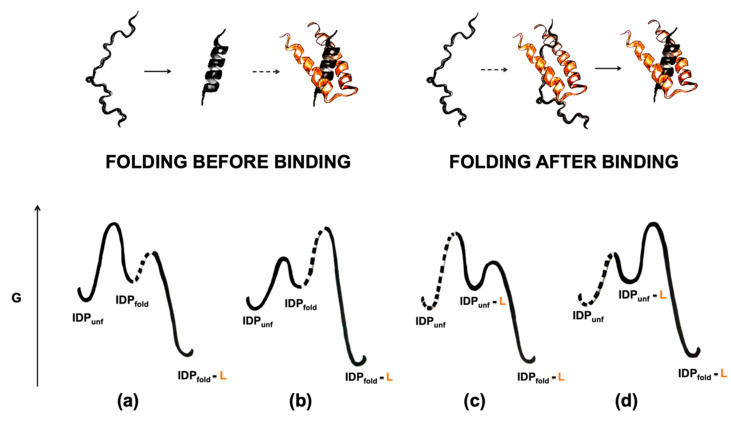
Conformational selection (Folding before binding) and Induced-fit (Folding after binding) models and related free energy diagrams. The ligand (L) and the intrinsically disordered protein (folded, IDP_fold,_ and unfolded IDP_unf_) are colored in black and orange respectively. A plausible energy diagram arising in different kinetic conditions is depicted in panels (**a**–**d**), in all cases the bimolecular binding reaction is highlighted with dashed lines. The implications of different scenarios are discussed in the text.

Under such conditions, if the overall reaction mainly occurs through steps 1 and 2, this would correspond to an induced-fit model, whereby binding precedes the conformational change [43]. Alternatively, if binding takes place through pathways 3 and 4, the folded and unfolded conformations of the IDP are in pre-equilibrium and the reaction resembles a conformational selection, formally similar to the MWC model [44]. Furthermore, a different behavior might be observed if, depending on reaction conditions, either folding or binding act as a rate-determining step [45]. Figure 2 summarizes a plausible free energy diagram of the induced-fit and MWC scenarios invoking folding or binding as different rate-limiting steps. Importantly, it has been shown that a possibility to discriminate between these different scenarios arises when performing experiments in pseudo-first order conditions with either the IDP or the ligand [12,13]. However, what are the main differences expected in the interpretation of Φ value analysis in IDPs in these different cases?

### 4.1. Folding before Binding

In the case of folding before binding, the IDP populates the bound conformation even in the absence of a ligand. When encountering the ligand, there is a shift in equilibrium and folding is promoted. In this case, folding might be slower than binding (Figure 2a) or vice versa (Figure 2b).

If folding were slow, when binding is experimentally carried out by mixing the IDP with increasing concentrations of ligand, the observed rate constant would be equal to

(9) $$k_{obs}=k_{F}$$

Thus, the observed rate constants would be independent on ligand concentration. In these cases, because the reaction would be limited by folding, the measured Φ values should resemble what is typically observed in globular folding studies, with clusters of native-like structure in the transition state. While theoretically possible, however, because IDPs are generally found to fold quite rapidly [15,22,24,26,42] and folding rate constants are normally correlated with the topological complexity of the molecule, a slow folding step preceding binding is expected to be very rare, especially in the case of IDPs displaying a simple topology in their ordered conformation.

An alternative, and more likely, possibility implies folding of the IDPs to be faster than binding (Figure 2b). In this case, the apparent rate constant would result from a combination of microscopic rate constants as formalized below:

(10) $$k_{on}^{app}=k_{on}\frac{1}{1+K_{D-N}}$$

where *k_on_* represents the bimolecular rate constant between the folded state and the ligand and *K*_D-N_ the unfolding equilibrium constant. Importantly, in these cases, the association between two fully folded entities would essentially limit the reaction rate. Thus, it would be expected that mutations purely affecting the folding of the IDP, i.e., mainly affecting *K_D-N_*, would generally lead to high values of Φ. Whereas variations located at the interface between the IDP and its partner should return more fractional values.

### 4.2. Folding after Binding

In many cases, the folding mechanism of IDPs has been found to occur via an induced fit scenario [12,13,14,22,45,46,47]. Under such conditions, folding occurs after the formation of an initial ‘encounter complex’, characterized by the binding between the IDP in its disordered conformation and the ligand. In these cases, if the microscopic rate constant of folding *k_F_* were slower than the microscopic rate constant of dissociation of the initial encounter complex (*k_off_*) (Figure 2c), the overall apparent association rate constant would be equal to

(11) $$k_{on}app=k_{on}\frac{1}{1+\frac{k_{off}}{k_{F}}}$$

where *k_on_* and *k_off_* represent respectively the rate constants of association and dissociation rate constant for the initial encounter complex, and *k_F_* the folding rate constant. On the other hand, if the folding rate constants were larger than the microscopic *k_off_* of binding (Figure 2d), the apparent association rate constant will be equal to the microscopic *k_on_*. These two different scenarios may lead to very different clusters of Φ values. In fact, in the latter case, the protein is expected to be largely disordered in the transition state. Thus, the Φ values calculated from variations affecting the folding of the IDP are expected to cluster around low values. An example of this case is nicely illustrated by the interaction between TAZ1 and TAD-STAT2 [48]. Conversely, in the former case, the main rate limiting barrier is associated to a folding step and Φ values would therefore approach the values of genuine folding probes. Accordingly, observation of high values of Φ, as observed for example in the binding between KIX and the transactivation domain of KIX [28], may suggest that *k_F_* < *k_off_* and allows to exclude the presence of a fast folding step occurring after slow binding.

## 5. The Residual Structure of IDPs

One of the critical assumptions of the Φ value analysis lies in a negligible effect of the mutations on the structure and free energy of the denatured state. In particular, it should be noticed that, if the effects on the denatured state are considered, Equations (1) and (2) should be rearranged to:

(12) $$\Delta \Delta G_{D-N}=\Delta G_{N\prime -N}-\Delta G_{D\prime -D}$$

(13) $$\Delta \Delta G_{D-TS}=\Delta G_{TS\prime -TS}-\Delta G_{D\prime -D}$$

where Δ*G_N’-N_*, Δ*G_TS’-TS_* and Δ*G_D’-D_* denote the changes in stability upon mutation of the native, transition and denatured state respectively. When the term Δ*G_D’-D_* tends to zero, the effect of the mutation on the denatured state is negligible and the Φ value will tend to its simpler value (Figure 1). On the other hand, a pronounced effect of the mutation on the denature state may distort the significance of the Φ values and more complex scenarios to interpret their values should be taken into account. The typical signature of these cases lies in the presence of so-called unusual values of Φ, i.e., negative values and/or values exceeding 1 [49]. These values are normally less than 10% and are expected to remarkably increase in frequency in the presence of a detectable effect the Δ*G_D’-D_* upon mutation.

Ever since the discovery of IDPs, it has been observed that these proteins in isolation may retain a considerable amount of embryonic structure [50,51,52,53], which may considerably vary from case to case and can be experimentally addressed with different experimental techniques such as NMR or SAXS [54,55,56]. Therefore, since mutagenesis may potentially perturb these structures, when evaluating IDPs as candidates for the Φ values analysis, it is important to take into account these possible effects. In fact, if the mutations on the IDPs affect their structure in isolation, there will be an effect in the measured induced folding Φ values.

As recalled above, a signature of the presence of a detectable effect on the denatured state upon mutagenesis is represented by the presence of unusual values of Φ [49]. Remarkably, none of the Φ values analysis reported to date appear to display this feature, indicating that the residual structure in the free state of the IDPs tested to date is relatively robust to site-directed mutagenesis and, in agreement with what observed in globular proteins, the value of ΔG_D’-D_ is negligible. This finding reinforces the significance of Φ values analysis, indicating that the effects of mutagenesis on the unbound state of IDPs do not represent a relevant complication in performing this type of experiments. More experimental work to further substantiate this finding is surely welcome.

## 6. Folding Pathway Malleability of IDPs

The inherent principle of Φ values analysis lies in providing structural information by monitoring the effect of perturbations on the pathway. Implicit in this premise, however, it is assumed that the perturbation per se does not significantly distort the overall mechanism [57]. In fact, if the mutation would cause a substantial de-routing of the folding pathway, a comparison between the transition state of the mutant and wild-type proteins would be prevented and the structural information in the Φ values analysis would therefore be compromised.

The analyses of the binding-induced folding of different IDPs have suggested this class of proteins to display malleable pathways that are partly dictated by their physiological partner [22]. In particular, in IDPs, folding may occur via heterogeneous nucleation, whereby the interactions stabilizing the transition state are directly established with the interacting ligand [22,24,30]. Thus, at variance with the robust mechanisms observed in the case of globular proteins, folding of IDPs tends to occur via a ‘templated folding’ mechanism, whereby the structure of the transition state is dictated by the nature of the interacting partner. However, is the folding of IDPs robust enough to be subjected to Φ values analysis?

**Figure 3:**
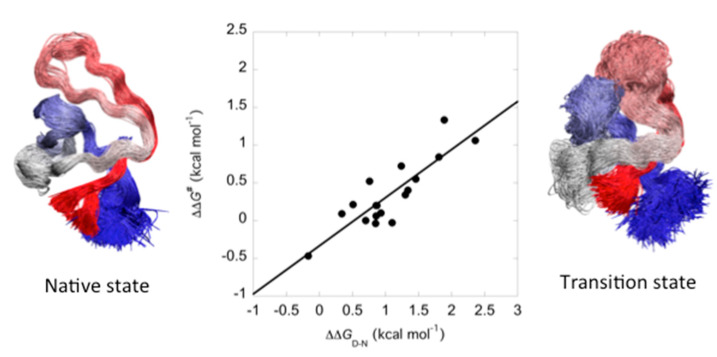
Linear Free Energy Relationship (LFER) plot in protein folding. The data refer to the Φ value analysis of the SH3 domain from Grb2 (81). The structure of the native state, obtained by NMR, and the transition state, calculated by using restrained molecular dynamic simulations, are reported on the left and on the right of the plot, respectively. As explained in the text, linearity in LFER plots is a hallmark of the nucleation-condensation mechanism and represents a common feature of globular proteins. By following this scenario, the transition state is a distorted version of the native state, as nicely exemplified by the data reported in the figure.

In chemistry, a classic procedure to infer the structural features of a reaction transition state is to perform a linear free energy relationship analysis (LFER) [58]. By applying this method, the changes in free energy of the transition states are related to changes in equilibrium free energies. If this relation returns a linear behavior, it may be concluded that the transition state is similar in structure to the ground state, with a position along the reaction coordinate that is proportional to the slope of the observed correlation, classically denoted as α. In the limit condition in which α equals 1, the structure of the transition state is identical to that of the product. Implicit in this view, the linearity of LFER plots is a clear signature of the robustness of the pathway. In fact, if the transition state displays similar native-like features, even when the stability is remarkably perturbed, it follows that it maintains the same structure. An example of a LFER analysis in protein folding is reported in Figure 3.

Remarkably, whilst the folding of IDP was found to be malleable to changes in the interacting partner [22,24,30], different studies performed to date appear to confirm that also this class of proteins returns linear LFER plots [12,27,28,30,59]. This finding suggests that the conservative mutations routinely employed to perform Φ values analysis do not cause a substantial rerouting of the pathway and represent an important internal control on the validity of Φ values analysis in IDPs.

## 7. Additional Tests—Double Mutant Cycle and Φ−Φ Plots

A powerful method to investigate the energetic communication between different sites within a protein is represented by the use of double mutant cycles [60]. This experimental approach has been introduced to detect and quantify interaction networks that modulate the allosteric communication within the protein moiety. In summary, double mutant cycles are based on the principle that when and if two amino acidic side chains within a protein are not energetically connected, the effects on mutating them will be additive.

As outlined above, a major complication when performing a Φ values analysis on IDPs arises from the possible effect of the individual mutations on either the structure of the IDP in isolation or on its folding pathway (i.e., de-routing effects) [22]. We suggest that a plausible test to reinforce the validity of Φ values analysis in IDPs could be represented by the usage of double mutant cycles. In fact, when and if the structure of the IDP in isolation or its folding pathway are robust to single aminoacidic substitutions, the changes in free energies induced by mutagenesis should be additive.

In practice, by considering a system P-AB (displaying two residues under investigation A and B), the measurement of the energetic contributions of these residues to the binding-induced folding of the IDP might be explored by producing the deletion mutants P-A, where B is varied; P-B, where A is varied; and P, where both residues are mutated [60]. By applying equation 6 and 7 to these three constructs, it may be observed that

(14) $$\Delta \Delta G_{Free-TS}^{P}=\Delta \Delta G_{Free-TS}^{PA}+\Delta \Delta G_{Free-TS}^{PB}$$

(15) $$\Delta \Delta G_{Free-Bound}^{P}=\Delta \Delta G_{Free-Bound}^{PA}+\Delta \Delta G_{Free-Bound}^{PB}$$

**Figure 4:**
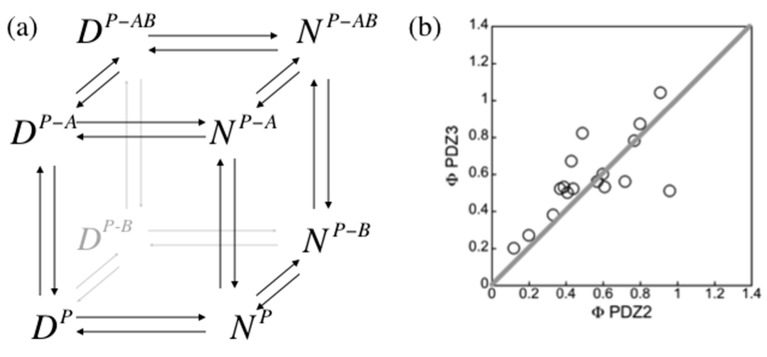
Additional tests for the Φ value analysis. (**a**) exemplifies an energy diagram summarizing the double mutant cycle approach. In analogy to classical protein folding, the reactant and product are referred to as the D (denatured) and N (native) states respectively. Analogously, in binding, these could represent the free and bound state. (**b**) Φ−Φ plot of two homologous PDZ domains (data from [61]). The correlation between the observed values is a signature of conservation of the folding mechanism and provides per se an additional validation of the Φ value analysis.

Inspection of Equations (14) and (15) reveals that when two residues do not interact energetically, the changes in free energy upon their mutations should be additive (Figure 4a). Analogously, in the absence of de-routing effects, double mutant cycles in IDP systems should result in additive changes in free energy on both the transition and ground state. This experiment would represent an informative test to probe the robustness of folding pathways in IDPs. If, in fact, the residual structure of the IDP is not substantially perturbed by mutations, the kinetic effects of two single variants should be additive in a double mutant and the sum of the two effects should be observed.

An informative method to compare folding pathways of different proteins lies in plotting their Φ values at homologous positions [62] (Figure 4b). This type of analysis, named Φ−Φ plot, can be carried out on proteins sharing the same structure but displaying different amino acidic compositions (such as homologous proteins) [61,63] and/or sequence connectivity (such as circular permutants) [64]. Importantly, it has been shown that the linear correlations in Φ−Φ plots represent per se a strong validation of the Φ value analysis [62]. In fact, linear Φ−Φ plots may be obtained only when (i) two proteins display a similar folding pathway and (ii) there is relevant structural information in the measured Φ values. In the case of IDPs, Φ−Φ plots may be carried out on homologous proteins, sharing a similar structure when bound to their physiological partner, as for example in the case of the viral proteins NTAIL and XD from different Paramoxyviruses [65].

Whilst both double mutant cycles and Φ−Φ plots would represent compelling tests to support Φ values analysis in IDPs, no study has performed a double mutant cycle, and only one Φ-Φ plot leading a linear correlation, to date, has been performed [11]. We call for additional work in this perspective, which can be critical in providing structural information about the mechanism of folding upon binding of IDPs, as well as in providing a direct validation of Φ values analysis per se.

## 8. Conclusions

The Φ values analysis represents the only experimental method offering the tantalizing possibility to address structurally metastable states. Nevertheless, in the case of IDPs, there are some inherent complications that demand additional care. With the specific purpose to help the experimentalist, we have summarized some of these key problems along with some considerations to avoid them. Future work on different IDP systems will be critical to understand the general rules of their folding mechanisms as well as the differences and similarities with the behavior observed in globular proteins.
